# Detection of Huntington’s disease decades before diagnosis: the Predict-HD study

**DOI:** 10.1136/jnnp.2007.128728

**Published:** 2007-12-20

**Authors:** J S Paulsen, D R Langbehn, J C Stout, E Aylward, C A Ross, M Nance, M Guttman, S Johnson, M MacDonald, L J Beglinger, K Duff, E Kayson, K Biglan, I Shoulson, D Oakes, M Hayden, The Predict-HD Investigators and Coordinators of the Huntington Study Group

**Affiliations:** 1 University of Iowa, The Roy J and Lucille A Carver College of Medicine, Iowa City, Iowa, USA; 2 Indiana University, Department of Psychology, Bloomington, Indiana, USA; 3 University of Washington, Department of Radiology, Seattle, Washington, USA; 4 Johns Hopkins University School of Medicine, Department of Psychiatry, Division of Neurobiology, Baltimore, Maryland, USA; 5 Park Nicollet Clinic, Department of Neurosciences, St Louis Park, Minnesota, USA; 6 Center for Movement Disorders, Markham, Ontario, Canada; 7 Massachusetts General Hospital, Neuroscience Center, Boston, Massachusetts, USA; 8 University of Rochester, Clinical Trials Coordination Center, Rochester, New York, USA; 9 University of Rochester Medical Center, Department of Biostatistics, Rochester, New York, USA; 10 University of British Columbia, Medical Genetics, Vancouver, British Columbia, Canada; 11 Huntington Study Group, Rochester, New York, USA

## Abstract

**Objective:**

The objective of the Predict-HD study is to use genetic, neurobiological and refined clinical markers to understand the early progression of Huntington’s disease (HD), prior to the point of traditional diagnosis, in persons with a known gene mutation. Here we estimate the approximate onset and initial course of various measurable aspects of HD relative to the time of eventual diagnosis.

**Methods:**

We studied 438 participants who were positive for the HD gene mutation, but did not yet meet the diagnostic criteria for HD and had no functional decline. Predictability of baseline cognitive, motor, psychiatric and imaging measures was modelled non-linearly using estimated time until diagnosis (based on CAG repeat length and current age) as the predictor.

**Results:**

Estimated time to diagnosis was related to most clinical and neuroimaging markers. The patterns of association suggested the commencement of detectable changes one to two decades prior to the predicted time of clinical diagnosis. The patterns were highly robust and consistent, despite the varied types of markers and diverse measurement methodologies.

**Conclusions:**

These findings from the Predict-HD study suggest the approximate time scale of measurable disease development, and suggest candidate disease markers for use in preventive HD trials.

Huntington’s disease (HD) is a dominantly inherited disease for which predictive testing can inform whether, but not precisely when, the disorder will manifest itself. The onset ages for HD have non-linear inverse relationships with the number of polyglutamine repeat sequences in the gene mutation so that younger diagnosed patients tend to have longer repeat lengths.^1^ ^2^ Current guidelines for genetic counselling recommend against using predictions of diagnosis age, in part because no curative or preventive treatments are available, and also because such estimates have large confidence intervals,^3^ making it impossible to accurately predict when the clinical disease will become manifest. Nonetheless, DNA based prognostic stratification can play a useful role in characterising attributes at the group level among those with various lengths of the HD CAG expansion. For example, in samples of individuals with the HD CAG expansion but not yet meeting diagnostic criteria for HD, at least two studies have indicated that minor motor signs are evident several years prior to diagnosis,^4^ ^5^ and more than 20 studies have shown measurable cognitive impairments.^6^^–^11 Psychiatric disturbances are prevalent prior to diagnosis of HD.^12^^–^16 At least seven studies have evaluated volumetric MRI in CAG expanded individuals prior to diagnosis,^17^^–^21 with a recent report suggesting that striatal volume loss is evident at least 9–11 years prior to estimated onset.^22^ Studies using functional MRI have shown reduced activation patterns in the basal ganglia^23^ and cingulate cortex^24^ in the absence of volumetric loss in prediagnosed individuals, suggesting that abnormalities in cell function may be detectable prior to cell death. Data from animal studies are consistent with these findings and indicate that receptor changes and electrophysiological changes clearly precede the onset of behavioural phenotype(s) in HD transgenic mouse models.^25^ Moreover, a few recent longitudinal studies have validated cross sectional findings of prediagnosis disease markers.^12^ ^22^

To reach the goals of prevention and intervention prior to clinical manifestations of HD, we must build on this evidence of prediagnostic changes in the brain and behaviour and attempt to further infer the longitudinal course of changes prior to diagnosis. For example, testing of effective preventive treatments may only be practical once it is possible to measure early disease progress prior to manifest clinical debilitation, and then to track these measures in response to candidate interventions. In this report, we have utilised estimates of time until diagnosis to examine early declines in HD biomarker and refined clinical marker changes. We used a large cohort of prediagnosed participants from the Predict-HD study, all of whom had verified and measured CAG expansions for HD.

## METHODS

### Participants

Predict-HD is a longitudinal study of individuals known to be at genetic risk for HD. This report is based on baseline data from all participants (n = 449 with CAG expansions in the HD gene) who were enrolled from September 2002 until October 2004. Participants were primarily Caucasian (96%), right-handed (89%), married (70%) employed (77%) females (64%) with an average age of 42.1 (SD 9.9) years. Ninety per cent had at least a high school education; average years of education for this sample was 14.5 (SD 2.6) years.

Participants were recruited at 17 sites in the USA, four sites in Canada and three sites in Australia (see Paulsen and colleagues^27^ for an overview of recruitment procedures). Study activities were reviewed and approved by institutional review boards at all study and data processing sites. Participants underwent informed consent procedures and signed consents for both participation and to allow de-identified research data to be sent to collaborative institutions for analyses.

To ascertain a comparison cohort, sites were asked to enrol a participant with a CAG repeat length in the reference range (eg, <30 repeats) for every six participants enrolled with a CAG repeat length in the HD range (ie, ⩾39 repeats). Inclusion criteria required participants to have previously undergone voluntary genetic testing for the presence of the CAG expansion in the HD gene, *huntingtin*, on the short arm of chromosome 4. Exclusion criteria included: (a) clinical evidence of unstable medical or psychiatric illness; (b) history of other central nervous system disease or event, such as seizures or head trauma; (c) pacemaker or metallic implants; and (d) prescribed antipsychotic or phenothiazine derivative antiemetic medications within the past 6 months. Other prescribed, over the counter and natural remedies were not restricted. Although the intent was to enrol only participants who were prediagnosed, provisions were made to enrol the few otherwise eligible individuals who seemed to be having HD symptoms if those individuals had not been diagnosed as having HD.

### Procedure

Participants underwent annual study visits at local sites, including blood draws, a neurological examination, cognitive assessment, psychiatric and psychological questionnaires, and brain MRI. Only a subset of data collected in these assessments is presented in the current report. Data and samples were sent from local sites to the Predict specialty sites, including blood samples to Harvard University for DNA analyses of CAG repeat lengths, cognitive assessment data to Indiana University at Bloomington for scoring and quality control, and MRI datasets to the University of Washington in Seattle for volumetric analyses of the caudate nucleus and putamen. These data, as well as general case report forms, were then forwarded to the Clinical Trials Coordination Center at the University of Rochester, where data were collated and missing data were queried. Finally, data sets were sent to the University of Iowa for construction of the comprehensive database and statistical analysis.

### Data included in the current report

#### Motor scores

The Unified HD Rating Scale^26^ motor examination consists of 15 primary items administered by movement disorder specialists who had undergone reliability training for the Predict-HD study. Total motor scores in this sample ranged from 0 to 34 (mean 5.53 (SD 5.40)) of a possible 124 points.

#### HD diagnostic confidence rating

After performing the motor examination, the movement disorder specialist assigned a diagnostic opinion, with level of certainty, according to the following scale: 0 = normal (no abnormalities); 1 = non-specific motor abnormalities (less than 50% confidence that the participant has manifest HD); 2 = motor abnormalities that may be signs of HD (50–89% confidence); 3 = motor abnormalities that are likely signs of HD (90–98% confidence); or 4 = motor abnormalities that are unequivocal signs of HD (⩾99% confidence). This scale is based on the currently accepted formal diagnostic process, which requires the emergence of an otherwise unexplained extrapyramidal movement disorder in someone at risk for HD. Thus the rating does not directly reflect the presence of cognitive or psychiatric manifestations of HD, which may also be present. By consensus, a rating of 4 is considered to be the point at which HD diagnosis is made. As we were focused on HD development before the point of diagnosis, we excluded 11 participants from this analysis who had a diagnostic rating of 4 on their initial examination. Of the remaining 438 participants analysed, confidence ratings were as follows: 148 (34%) rated 0, 198 (45%) rated 1, 68 (16%) rated 2 and 24 (5.5%) rated 3.

#### Determination of CAG length

A previous voluntary decision to undergo HD gene testing was a prerequisite for entry into the Predict-HD study and clinical predictive testing results were confirmed by CAG repeat lengths obtained via PCR, as described elsewhere.^27^ For the study sample, CAG expansions ranged from 39 to 50, with a mean of 42.3 (median 42).

#### Estimation of time to HD diagnosis

**Figure 1:**
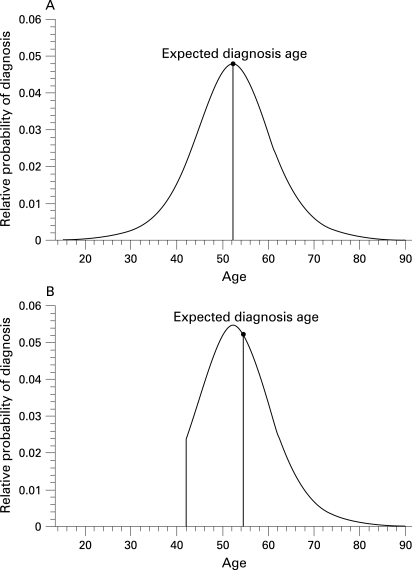
(A) Example estimated probability density function for age at diagnosis of Huntington’s disease (HD), calculated at birth, for a person with a CAG expansion length of 42. Line and circle illustrate the expected age of diagnosis (mean of the distribution), which is 52.2 years. (B) Example estimated probability density function for age at HD diagnosis, for a person with a CAG expansion length of 42, given that the person has reached age 42 years without being diagnosed. Distribution from (A) is truncated at the present age and the remaining area under the curve is rescaled to equal 1. Line and circle illustrate the expected age of diagnosis for this adjusted distribution (ie, the mean of the age conditional distribution), which is 54.5 years. Therefore, the expected time until diagnosis is 54.4–42 = 12.4 years.

To examine relationships between proximity to HD diagnosis and motor, brain volumetric and cognitive measures, we used a CAG based prognostic model developed^3^ on the basis of nearly 3000 subjects. This survival formula can be transformed to a probability distribution for age of diagnosis that depends on both the subject’s CAG expansion length and current age (fig 1A). We give details, including assumptions and transformations to estimate years until diagnosis, in appendix A online. Briefly, in order to estimate mean time until clinical diagnosis, we truncate the probability distribution to account for the fact that a subject has reached their current age without yet receiving a diagnosis. We then calculate the mean of this revised distribution (fig 1B). The study sample had a mean estimated time to diagnosis of 13.9 (SD 6.7) years.

#### Cognitive assessment

Cognitive testing included nine paper and pencil tests from the well established clinical neuropsychological literature and nine computerised tasks developed specifically for Predict-HD which were administered on standardised specially constructed pc based computer systems. All study personnel who administered the cognitive battery underwent in-person training until they reached the criterion for standardised administration, followed by annual reliability checks. Only four of 18 tests were used in the current report. Tests were selected to represent varying components of brain processes (motor speed, timing, memory and sensory processes).

#### Motor speed

The Speeded Tapping Test^28^ is a computerised test of finger tapping speed that requires a participant to press keys on a response box as quickly as possible. The value analysed for this study was the average tapping rate. Previous research has suggested that motor speed may be maintained by the motor/premotor circuit.

#### Self-paced timing consistency

Participants tapped on a single key in time to a series of 11 evenly paced tones, after which they had to continue tapping at the same pace for another 31 taps. To capture the consistency in self-paced movements, we computed the mean of the SD for six artefact free trials. Previous research indicates that the dorsolateral circuit may be involved in time discrimination.

#### Verbal learning/memory (word list learning)

A traditional list learning task, the Hopkins Verbal Learning Test-Revised,^29^ was used. For three trials, participants listened to the same 12 item word list and had to immediately recall aloud the words read by the examiner. Human learning and memory is often associated with frontal temporal circuitry.

#### Odour recognition

The University of Pennsylvania Smell Identification Test^30^ is a 40 item, four choice scratch and sniff test. For the model analyses, we examined the number of correct identifications out of a possible 40. Animal and human research suggests that olfactory performances may be associated with the lateral orbitofrontal circuit.

#### MRI measures

All scans for this project were obtained using a standard protocol designed to optimise visualisation of the basal ganglia. We obtained an axial three-dimensional volumetric spoiled gradient echo series, with a flip angle of 20°, TE = 3, TR = 18, FOV = 24 cm, 124 slices at 1.5 mm/slice, matrix 256×192, ¾ phase FOV and NEX = 2. All sites used a General Electric 1.5 T scanner (with one exception of a site using a 1.5 T Siemens scanner). A single rater, who had been trained to >0.95 for inter-rater reliability with one of the authors (EA), was blinded to participant characteristics and completed all measurements for the study. Measurements were made by manually drawing boundaries of the caudate (head and body) and putamen, as previously described,^17^^–^21 and total striatal volumes were calculated based on the number of identified pixels. The data were analysed in the order received. MRI data from 261 of the Predict participants had been analysed at the time of this report. The MRI sample is smaller because of a lag induced by the extra processing steps needed to produce the volumetric data and does not reflect any other known sampling bias. Striatal volumes ranged from 9.2 to 25.4 ml (mean 16.9 (SD 3.0) ml).

### Statistical analyses

The primary goal was to use the study’s baseline data to determine the relationship of estimated years to diagnosis with biological (brain volume) and refined clinical (cognitive, motor) measures. Because we wanted to test for non-constant slopes in these relationships, we fitted flexible non-linear models to estimate them. Specifically, we fitted least squares models based on cubic restricted spline transforms of the estimated time to diagnosis. We used predefined knot locations to improve the validity of statistical inference.^31^ These spline models typically have an advantage over polynomial models in that, using similar degrees of freedom to test deviation from strictly straight line regression, the allowable curvature shapes more plausibly reflect real physical relationships. To protect against over fitting, we restricted our choices to splines that added either 1 or 3 extra degrees of freedom for potential non-linearity and, between these, selected the model with better adjusted R square (variance explained) estimate. All models were adjusted for participant age, gender and, in the case of cognitive outcomes, years of education and estimated premorbid IQ (as reflected by Anart test scores). We made these adjustments by including the additional terms as conventional multiple regression covariates. As a secondary analysis, clinical motor score was also controlled in order to better interpret the results of psychomotor test analyses.

We performed residual diagnostics on all models. We found no need to remove more than one outlier in any instance. Such outliers had small effects on details of the curve fit, but there was no occasion where they would have changed the statistical significance of the models.

Spline model results are not readily interpreted in terms of traditional regression coefficients. Instead, we present graphs of the fitted curves, along with partial adjusted R square (“variance explained”) contributed by the expected time to diagnosis and the p values for both the total and non-linear contributions of expected time to diagnosis. Statistical significance attached to the non-linear contribution reflects the alternative hypothesis that the mean rate of marker progression changes with estimated time to diagnosis.

**Table 2:** Non-linear model fits for estimated age at diagnosis associations adjusted for motor scores

Variable	n	Adj R^2^	p Value	Non-linear df	Non-linear p value
Striatal volume	261	0.20	<0.0001	1	0.002
Speeded finger tapping rate (mean)	408	0.09	<0.0001	3	<0.01
Consistency in self-timed finger tapping	410	0.14	<0.0001	3	<0.01
Word list learning (HVLT)	425	0.05	<0.0001	1	0.10
Odour identification	424	0.07	<0.0001	1	<0.01

Adj R^2^  =  variance accounted for by estimated years to diagnosis of Huntington’s disease (HD) after accounting for covariates (see statistical methods section) and degrees of freedom used for non-linear fit.

HVLT, Hopkins Verbal Learning Test; Non-linear df, degrees of freedom for non-linear fit; non-linear p value, p value for non-linear element of estimated years to HD diagnosis fit.

## RESULTS

**Figure 2:**
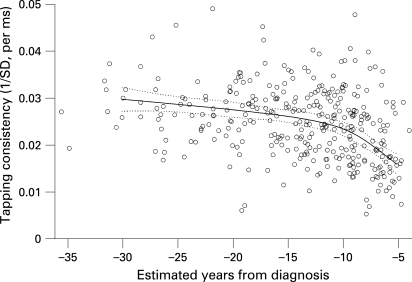
Scatterplot of self-timed tapping consistency data and estimated years from diagnosis of Huntington’s disease (HD). The fitted spline relationship, including 95% confidence limits for the fit, is superimposed. The plotted data points are adjusted for age, gender, education and estimated IQ by linear regression and standardised to a female with mean values of the other values. As detailed in table 1, the adjusted per cent variance explained (R^2^) for this plot is 0.20.

A detailed example of one of our analyses is shown in fig 2 where we illustrate the relationship between estimated time to diagnosis and timing consistency measured by the self-paced tapping task. The individual data points are included to give a clear sense of the extent of model fit. In this case, approximately 20% of the subject to subject variability in performance is explained (table 1). Visually, the non-linear trend captured in the model is apparent in the raw data. Of course, it is also evident that there is additional person to person variability that our model cannot account for.

**Table 1:** Non-linear model fits for associations with estimated age at diagnosis

Variable	n	Adj R^2^	p Value	Non-linear df	Non-linear p value
Motor examination score	438	0.15	<0.0001	3	<0.0001
Striatal volume	261	0.23	<0.0001	1	<0.001
Speeded finger tapping rate (mean)	408	0.14	<0.0001	3	<0.0001
Consistency in self-timed finger tapping	410	0.20	<0.0001	3	<0.0001
Word list learning (HVLT)	425	0.09	<0.0001	1	<0.01
Odour identification	424	0.10	<0.0001	1	<0.0001

Adj R^2^ = partial adjusted variance accounted for by estimated years to diagnosis to Huntington’s disease (HD) after accounting for covariates (see statistical methods section) and degrees of freedom used for non-linear fit.

HVLT, Hopkins Verbal Learning Test; Non-linear df, degrees of freedom for non-linear fit; non-linear p value, p value for non-linear element of estimated years to HD diagnosis fit.

**Figure 3:**
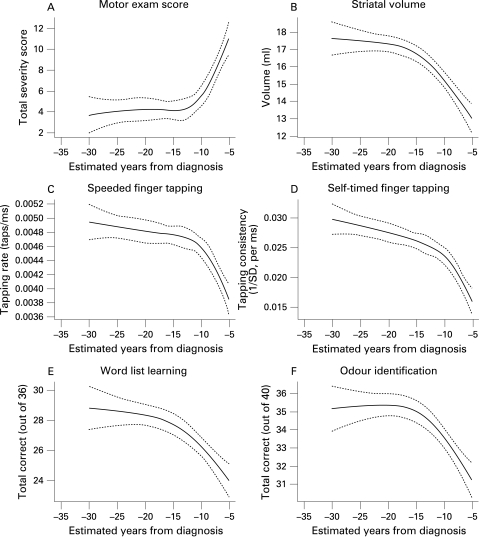
Relationship between estimated years to diagnosis of Huntington’s disease (HD) and various other measures. Solid line plots the predicted response; broken lines are 95% confidence limits for the estimated mean response. All relationships are adjusted to a female with mean levels of the other variables for which we adjusted our models (age = 41.2 years, education = 14 years, premorbid IQ).

The estimated relationships between years to diagnosis and the other outcomes are illustrated in fig 3. As can be seen from the figures, motor examination scores, odour recognition, striatal volumes and a wide range of cognitive performances show a consistent convergent pattern, suggesting that little impairment is detected prior to 15–20 years from estimated diagnosis. Then, after a short transition period, a fairly linear relationship is seen. Table 1 shows model results in terms of total variance explained by the expected time to diagnosis. In every instance, both the total relationship with estimated time until diagnosis and the non-linear component were highly significant. Variance explained is in the 10–20% range.

It is conceivable that motor deficits may affect performance on some of the cognitive tests. To determine whether other associations were redundant with the motor association, we recomputed the corresponding models after adjusting for motor score as an additional covariate (see table 2). Although the association strengths were somewhat weakened, consistent with partial confounding, the striatal and cognitive variables still showed substantial non-linear associations.

## DISCUSSION

Findings suggest converging evidence from varying markers for detectable abnormalities beginning between one and two decades before diagnosis of HD. The relationships between estimated years to diagnosis and motor scores, striatal volumes, odour recognition and cognitive measures were strikingly consistent. On this estimated time scale, all domains suggest a curvilinear pattern of disease commencement. This period of commencement is followed by more rapid and constant change in the last years prior to diagnosis. Our findings are consistent with previous reports suggesting an association of striatal volumes with estimated diagnosis based on CAG repeat.^17^ ^18^ ^22^ The current sample size is nearly 10 times larger than previous reports and thus serves both a confirmatory and refining role. Additionally, demonstration of similar estimated time lines for cognitive, sensory and motor changes is novel and significantly extends these findings. We have illustrated a pervasive pattern of change, occurring on the same time scale, which extends from biological measures such as striatal volumes to several different clinical aspects of the HD phenotype.

Our finding that associations between years to diagnosis and other variables remained, even after controlling for motor signs, strongly suggests that apparent cognitive and sensory dysfunction cannot be explained solely on the basis of emerging motor signs interfering with task performance. It is noteworthy that these associations remained, regardless of whether the assessment was laden with motor demands (speeded finger tapping, self-timed finger tapping) or not (verbal learning, odour identification). The consistency of associations between the estimated genetic time scale and these diverse cognitive measures suggests that multiple corticostriatal circuits are simultaneously involved in early disease, although further research is needed to more explicitly track the brain behaviour associations suggested here. Striatal volumes also remained curvilinearly related to estimated years to diagnosis after controlling for motor signs. The analyses therefore indicate that neither cognitive nor striatal measurements are wholly redundant with the clinical motor examination. It follows that all of these can likely be combined and leveraged to improve the accuracy of individualised prognosis. Many of these markers may be candidate surrogate endpoints (bearing in mind that promotion from candidate to true surrogate is exceedingly difficult).^32^ However, markers that cannot meet criteria for this elusive role can still be useful for risk stratification—which is still quite valuable for increasing clinical trial power in a rare disease*.* Quantitative estimation of the impact of these markers in either role (longitudinal outcome or baseline stratification) is a substantial topic in its own right. In the near future, we will address the details of using baseline Predict-HD findings for increased statistical power and trial design efficiency in a separate manuscript.

There are some important caveats to consider when interpreting these findings. Firstly, the cross sectional associations require longitudinal validation. With planned follow-up periods of up to 7 years, the Predict-HD study will eventually yield appropriate data to attempt this validation. Furthermore, the study was designed to eventually yield a sufficient sample of individuals who become diagnosed, allowing CAG based estimates of average diagnosis age to be validated or refined. Despite excellent goodness of fit in the original model derivation,^3^ the time scale discussed in the current article remains provisional until longitudinal study is complete.

Even if the time scale is accurate, estimated time to diagnosis is clearly an imperfect proxy for actual time to diagnosis, which is of course unknown in these subjects. Although relationships based on estimated time to diagnosis cannot perfectly represent the relationship that would be seen if true years to diagnosis were known, it can be shown that an accurate expected time proxy leads to approximations providing useful bounds on the true average relationships. For example, the (essentially) uniformly concave or convex nature of each curve in fig 3 allows invocation of Jensen’s inequality^33^ to suggest that we are estimating an upper limit to the true time course of mean change. Further mathematical arguments beyond the realm of this paper also show that it is unlikely that this upper bound dramatically overestimates the true mean time of onset of these deficits. Hence while resisting the temptation to over interpret these curves, we feel comfortable in our claim that they are strong evidence that detectable deficits begin between 10 and 20 years before HD diagnosis in the average CAG expanded subject. A more detailed and mathematical exposition regarding the limits of interpretability for this class of prognostic model will also be forthcoming in a separate report.

Which of the outcomes described here is the strongest marker of developing HD? Again, we must remember that the models we have presented actually reflect the relationship between these markers and another known marker—CAG repeat length (corrected for age). While the relative strengths of these relationships provide a tentative basis for judgment, they are not guaranteed to reflect the relative prognostic importance that we will eventually observe with actual clinical diagnosis times*.* Similarly, we cannot yet know to what extent each measure independently contributes prognostic information. For example, striatal volume shows the strongest relationship to estimated prognosis in our analyses. Given the well known central role of basal ganglia deterioration in HD, we will not be surprised if this measure remains the strongest individual predictor when we can analyse observed onset. Nonetheless, it is possible that its relative strength in the current analysis could be due to an especially tight CAG length association that does not necessarily translate into the best incremental independent prediction of true prognosis.

Our findings of cognitive, sensory, motor and striatal volumetric changes well before disease diagnosis are unequivocal, but it remains unknown whether these changes are of functional significance until our longitudinal data are acquired. Also, it is not known whether there are very subtle abnormalities, perhaps imperfectly reflected in these markers, prior to the evident upper limits that we have estimated here. Although not included in the current report, the Predict-HD study includes a comparison group of participants who are from HD families but are known not to have the CAG expansion. Once sufficient data from this sample become available, it may be possible to detect subtle abnormalities even earlier than the current report suggests.

Current hopes for reducing the burden of neurodegenerative disease rely on the idea of preventing disease onset and slowing progression so that people at risk may live out a longer portion of their lifespan as healthy, fully functioning individuals. For this to be possible, promising therapeutic agents must be tested for their ability to slow early disease progression. These cross sectional findings from Predict-HD indicate the approximate time scale of measurable disease development, and suggest disease markers which may be candidate surrogates for use in preventive trials. Furthermore, even markers that fail as treatment response surrogates will have great value for risk stratification in such trials. With validation and refinement through longitudinal study, it will be possible in just a few more years to confirm such markers, making it feasible to initiate the first preventive trials in individuals with the HD CAG expansion prior to functional decline and diagnosis.
